# Enhancing heart failure detection and patient engagement with the Maastricht Decompensation Questionnaire

**DOI:** 10.1007/s12471-025-01945-4

**Published:** 2025-02-27

**Authors:** Arno J. Gingele, Hans-Peter Brunner-La Rocca

**Affiliations:** Department of Cardiology, Maastricht University Medical Centre+, Maastricht, The Netherlands

Dear Editor,

Recently, we published the results of the Maastricht Decompensation Questionnaire (MDQ) trial, demonstrating that the MDQ effectively identifies decompensated heart failure (HF) patients using patient-reported signs and symptoms [1]. We appreciate the insightful commentary by Fiolet and Szymanski on our article [2].

They raised concerns about the use of HF nurses’ fluid status assessments as the reference standard. However, as the MDQ was administered during routine ambulatory evaluations, HF nurses had access to complementary data, including biochemical markers and regularly echocardiography, collected routinely as indicated. Thus, our standard was not solely based on physical examination.

While adding objective parameters may further improve risk prediction models, clinical practice often relies on history-taking, especially in remote consultations. The MDQ was designed to reflect this reality, and our study confirms its effectiveness.

To further improve its diagnostic accuracy, we are personalising the MDQ to align with individual patient profiles, enhancing sensitivity and specificity. Additionally, we are integrating features such as avatars and chatbots to improve patient engagement [3]. These innovations will be prospectively tested in a large cohort of HF patients.
